# Motor Imagery Learning Modulates Functional Connectivity of Multiple Brain Systems in Resting State

**DOI:** 10.1371/journal.pone.0085489

**Published:** 2014-01-17

**Authors:** Hang Zhang, Zhiying Long, Ruiyang Ge, Lele Xu, Zhen Jin, Li Yao, Yijun Liu

**Affiliations:** 1 School of Information Science and Technology, Beijing Normal University, Beijing, China; 2 Department of Biomedical Engineering, Peking University, Beijing, China; 3 C. Lauterbur Research Centers for Biomedical Imaging, Shenzhen Institutes of Advanced Technology, Chinese Academy of Sciences, Shenzhen, China; 4 National Key Laboratory of Cognitive Neuroscience and Learning, Beijing Normal University, Beijing, China; 5 Department of Psychiatry and McKnight Brain Institute, University of Florida, Gainesville, Florida, United States of America; 6 Laboratory of Magnetic Resonance Imaging, the 306th Hospital of PLA, Beijing, China; Cuban Neuroscience Center, Cuba

## Abstract

**Background:**

Learning motor skills involves subsequent modulation of resting-state functional connectivity in the sensory-motor system. This idea was mostly derived from the investigations on motor execution learning which mainly recruits the processing of sensory-motor information. Behavioral evidences demonstrated that motor skills in our daily lives could be learned through imagery procedures. However, it remains unclear whether the modulation of resting-state functional connectivity also exists in the sensory-motor system after motor imagery learning.

**Methodology/Principal Findings:**

We performed a fMRI investigation on motor imagery learning from resting state. Based on previous studies, we identified eight sensory and cognitive resting-state networks (RSNs) corresponding to the brain systems and further explored the functional connectivity of these RSNs through the assessments, connectivity and network strengths before and after the two-week consecutive learning. Two intriguing results were revealed: (1) The sensory RSNs, specifically sensory-motor and lateral visual networks exhibited greater connectivity strengths in precuneus and fusiform gyrus after learning; (2) Decreased network strength induced by learning was proved in the default mode network, a cognitive RSN.

**Conclusions/Significance:**

These results indicated that resting-state functional connectivity could be modulated by motor imagery learning in multiple brain systems, and such modulation displayed in the sensory-motor, visual and default brain systems may be associated with the establishment of motor schema and the regulation of introspective thought. These findings further revealed the neural substrates underlying motor skill learning and potentially provided new insights into the therapeutic benefits of motor imagery learning.

## Introduction

Motor skills in our daily lives could be acquired through execution or imagery learning [1]–[4]. This process of motor skill learning involves neural plasticity for functional reorganizations, thus it attracts more and more neuroimaging investigations through task or resting states [3]–[8].

Motor task-related changes measured from functional activities were mostly identified in primary motor cortex (M1), supplementary motor area (SMA), premotor area (PMA) for execution learning and visual cortex (e.g., the secondary visual cortex and fusiform gyrus) for motor imagery learning [2]–[4], [9]. Further results from functional connectivity and effective connectivity analyses indicated that functional reorganizations in the sensory-motor system after motor execution/imagery learning potentially subserved the establishment and consolidation of motor skills [10]–[12]. In addition, recent studies have mentioned several confoundings in interpretation of the findings associated with motor skill learning. For instance, baseline in the task state was not consistent during learning process, and changes in activation might simply reflect the changes in motor behavior when performing tasks after learning [6], [13]. Therefore, resting state has drawn much attention in the explorations of motor skill learning.

Spontaneous activity measured with blood-oxygen level-dependent (BOLD) in resting state appeared intrinsic and coherent in several distributed networks [14], [15]. These resting-state networks (RSNs) displayed spatial patterns with task-induced activations for sensory-motor, visual, auditory, attention and other cognitive processing in the corresponding brain systems [15], [16]. Therefore related neuroimaging explorations mainly focused on these sensory RSNs including sensory-motor network (SMN), the lateral/medial visual network (LVN/MVN), the auditory network (AN), and these cognitive RSNs including the dorsal/ventral attention network (DAN/VAN), the self-referential network (SRN) and the default-mode network (DMN) [17]–[19]. These RSNs remained consistent, irrespective of the variances in participants and the scanning sessions and may be integrated through resting-state functional connectivity [15], [20],

Using functional connectivity analysis, several fMRI studies have explored motor skill learning from resting state. Albert and his colleagues identified several motor-related resting-state networks (RSNs) and further revealed increased network strength of these RSNs after learning [7]. Ma et al. stressed a specific RSN in motor cortex and revealed changes of connectivity strength in right postcentral gyrus, bilateral supramarginal gyrus over the learning process [8]. Vahdat et al. incorporated behavioral measures into resting-state connectivity analyses and illustrated that changes of resting-state functional connectivity in motor-related RSNs were associated with the behavioural measures of perceptual and motor aspects after the visuomotor adaptation learning [13]. Furthermore, an association between structural alterations and functional connectivity variances was identified in the sensory-motor system after the dynamic balance training [21]. These findings primarily indicated that motor skill learning involved subsequent modulation of resting-state functional connectivity in the sensory-motor system. Nevertheless these explorations mostly focused on motor execution learning which mainly recruited the processing of sensory-motor information [22], [23]. Notably, no study investigated the motor imagery learning from resting state, although increasing evidences showed that motor imagery learning could improve the motor behavior and benefit the motor function rehabilitation [2]–[4], [24]. It remains unclear whether the modulation of resting-state functional connectivity also exists in the sensory-motor system after motor imagery learning.

To address this issue, we performed a fMRI investigation on motor imagery learning from resting state and investigated resting-state functional connectivity before and after the two-week consecutive learning. Based on previous studies, we first identified eight sensory and cognitive resting-state networks (RSNs) corresponding to the brain systems and further explored the functional connectivity of these RSNs through the assessments of connectivity and network strengths (see details in Methods). Since both sensory and cognitive processing were suggested to be recruited in motor imagery procedure [25], [26], we hypothesized that the modulation associated with motor imagery learning could be manifested in multiple sensory and cognitive brain systems. Our results verified this hypothesis and indicated that resting-state functional connectivity could be modulated by motor imagery leaning in the sensory-motor, visual and default brain systems.

## Methods

### Ethics Statement

The human fMRI experiment conducted in this study was approved by the Institutional Review Board of Beijing Normal University (BNU) Imaging Center for Brain Research, National Key Laboratory of Cognitive Neuroscience. All of the subjects gave written informed consent according to the guidelines set by the MRI Center of Beijing Normal University.

### Participants

Thirty-two right hand-dominant college students participated in the study, and several subjects subsequently dropped out due to health concerns or malfunctions of the equipment. At last, fourteen participants were included in the experimental group (seven males, mean age: 22±2 years) and twelve participants were included in the control group (five males, mean age: 24±2 years). Participants with histories of neurological disorders, psychiatric disorders, experience with typewriters, or any experience learning to play musical instruments were excluded. All participants passed the Edinburgh Handedness Inventory, the Movement Imagery Questionnaire and the Vividness of Movement Imagery Questionnaire [27], [28]. According to these questionnaires, we requested the participants to understand kinesthetic imagery, and to employ this imagery strategy during the whole experimental procedure.

### Experimental procedure

The experimental procedure included both resting- and task-state scans to allow for comparisons between the two states in the future. Therefore, the overall experimental procedure included a pre-rest scan, pre-task scans, an motor imagery learning period (experimental group) or a no-learning period (control group), a post-rest scan and post-task scans, and the present study mainly focused on the pre-rest and post-rest scans.

Outside the scanner, all the participants were instructed that from their index to little finger, each of the four fingers of their right hand represented a single digit number: one, two, three, and four. Thereafter, they went through a short period exercise to learn the 4 Hz rhythm with a metronome.

Before the learning period, the pre-rest scan, lasting for 10 minute, was used to establish a baseline condition for the examination. Subjects were instructed to keep their eyes closed, relax their minds and remain as motionless as possible. The pre-task scans, which included a motor execution and an imagery scan, were then performed. The two 4.5 min sessions (execution and imagery) were separated by a 5-min inter-session rest period. Each session consisted of four 30-s periods of executing/imagining the motor sequence, and these periods were interspersed with five 30-s rest periods. The assignment of scan order was counterbalanced across subjects. In each scanning session, a sequential finger movement task with the press sequence 4–2–3–1–3–4–2 was used. Subjects attempted to execute or imagine this sequence with their right hands at a self-paced rate of 4 Hz when PUSH was displayed on the screen and relaxed when REST was displayed on the screen. These indications were displayed to the subject via a mirror mounted on the head coil that reflected visually presented indications on a semi-transparent screen at the end of the scanner bore. Cushions inside the head coil were used to reduce head movement. The tapping sequence was performed with a four-button response pad, and the response pad was connected to a computer running the E-prime program (Psychology Software Tools, PA, USA) to record the responses. The participants were kept in the scanner for the whole procedure.

During the learning period, 14 motor imagery practice sessions were performed over 14 consecutive days to guarantee sufficient learning. Participants in the control group did not attend any learning sessions over the 14 days. Participants in the experimental group were trained under the supervision of the experimenter, and a cardboard box covered their right hands to prevent visual feedback. Participants were instructed to provide a qualitative description of their performance. The contents of the qualitative description were based on the Movement Imagery Questionnaire and included seven rating levels (1, very hard to feel; 2, hard to feel; 3, somewhat hard to feel; 4, neutral; 5, somewhat easy to feel; 6, easy to feel; 7, very easy to feel) [27]. No participants scored lower than 5. We further calculated the mean rating for each participant over the 14-day period and calculated the mean rating and the standard deviation for the 14 participants; the results (14 participants, mean rating: 5.9±0.7) ensured that the participants learned the motor imagery task. Each learning session consisted of two sections. One 15 min section was paced by the metronome, and the other 15 min section was paced by the participants themselves. Each section consisted of repetitive cycles of rest (30 s) and imagery practice (30 s). On the first two practice days, participants were paced at 2 Hz according to the behavioral results of the pre-execution task. This requirement was found to be important in a previous study and helped to ensure that participants could focus on establishing a representation of the sequence order [1]. From the third day onward, the pacing frequency was increased to 4 Hz to encourage participants to improve their tapping rates. Following the last learning session, all participants were scanned again using the same fMRI procedure employed for the pre-scans.

### fMRI data acquisition

Brain scans were performed at the MRI Center of Beijing Normal University using a 3.0-T Siemens whole-body MRI scanner. A single-shot T2*-weighted, gradient-echo, EPI sequence was used for functional imaging acquisition with the following parameters: TR/TE/flip angle = 3000 ms/40 ms/90°, acquisition matrix = 64×64; field of view (FOV) = 240 mm, and slice thickness = 5 mm with no inter-slice gap. Thirty-two axial slices parallel to the AC-PC line were obtained in an interleaved order to cover the entire cerebrum and cerebellum.

### Data Processing

Functional images from the pre- and post-resting scans were preprocessed for each subject. The time series were first realigned before being spatially normalized into standard stereotaxic space (the EPI template provided by the Montreal Neurologic Institute, MNI), re-sliced to 3×3×4 mm voxels, and smoothed with an 8×8×8 full-width-at-half-maximum (FWHM) Gaussian kernel using SPM8 software (Statistical Parametric Mapping; http://www.fil.ion.ucl.ac.uk/spm). Then, the group ICA, which includes dimension reduction by principle component analysis (PCA), ICA decomposition, and back reconstruction, was carried out with GIFT for circuitry exploration (http://icatb.sourceforce.net).

The preprocessed resting-state data from the pre- and post-rest scans were combined into a single group ICA analysis for each group (experimental and control group). Prior to the ICA analyses, two-step principal component analysis (PCA) was used to reduce the dimensionality of data, and the number of ICA components was set to 38 for both groups to preserve, on average, at least 99.99% of the original variance. Next, the data were decomposed by group ICA [29] using the Informax algorithm [30]. Spatially independent components were then back-reconstructed for all subjects. The components covering the sensory (SMN, LVN, MVN, AN) and cognitive (SRN, DMN, DAN, VAN) networks were identified visually and confirmed by selecting the components with the highest spatial correlations with the templates based on an independent dataset [19]. These components were converted to z-maps, and group t-maps were computed to determine the group spatial map and the corresponding spatial pattern of each RSN for the experimental/control group respectively (*p*<0.005, FDR corrected). Then, z-maps of each component were divided into four conditions for further analysis according to pre-/post-rest scans of the experimental/control group. In the subsequent analysis, connectivity strength and network strength were measured as in previous studies [7], [8]. Connectivity strength was measured as the z score of each voxel in a component z-map, which could assess the involvement degree of each voxel in the entire spatial maps of each RSN. Network strength was associated with the integrative connectivity of each RSN, and it was calculated as the sum of the z score of all voxels in a component z-map. To test the variance of connectivity strength after learning, the z-maps of each RSN were assessed with paired t-tests between the pre- and post-rest scans of the experimental/control group. Considering these variances may be scattered across several distributed voxels, which were induced by noises. A voxel-cluster threshold correction was used to control the Type I error rate in the whole-brain statistics, and this correction yielded an overall corrected alpha rate of *p*<0.05. The correction was determined from a Monte Carlo simulation and required a voxel-wise threshold of *p*<0.005 within a minimum 3D cluster of 41 contiguous significant voxels (minimum cluster volume = 1476 µl; FWHM autocorrelation estimate = 8.0 mm). Subsequently, the average z value of each RSN was measured as network strength. A repeated measures ANOVA model was employed to assess the changes in network strength according to the contrasts between the pre- and post-rest scans for the experimental/control group.

The consistency between the baselines of the experimental and control group were also tested. The resting-state data from the pre-scan for both groups were combined into a single group ICA. Prior to ICA, the number of ICA components was set to 38 to preserve, on average, at least 99.99% of the original variance, and the sensory and cognitive components were identified visually and confirmed via templates based on an independent dataset [19]. Subsequently, components were converted to z-maps, and then, connectivity and network strengths were assessed with two-sample t-tests for each component using a contrast between the experimental and control groups. No components showed significant variance of connectivity strength between the two groups during the pre-rest scan, and network strength of each component was also consistent between groups during the pre-rest scan (each *t*(24) <2.06 and *p*>0.05, see details in Figure S1).

## Results

**Figure 1:**
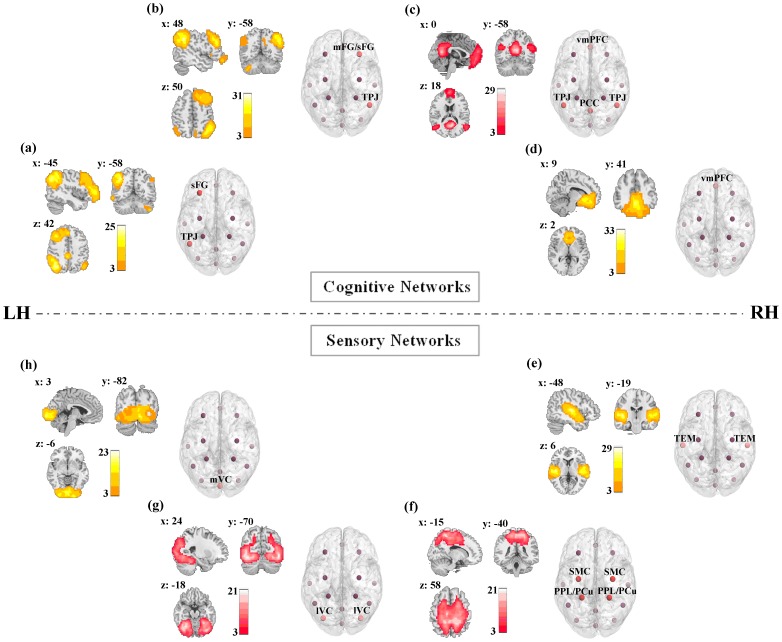
Coronal, sagittal, and axial views of the spatial maps and the corresponding spatial patterns of the cognitive and sensory networks in resting state. *a–h* represent the RSNs, including VAN, DAN, DMN, SRN, AN, SMN, LVN and MVN, for the experiment group respectively. Red nodes in each spatial pattern represented the regions significantly recruited in the RSN. The red spatial map indicated that the corresponding RSN has exhibited significant alterations of the resting-state functional connectivity after learning. Each map was the result of one-sample *t*-tests on the individual patterns that were identified using the combined data of pre- and post-rest scans, *p*<0.005, FDR corrected.

The RSNs identified as the components of group ICA analysis were shown in Figure 1. Regions of these components exhibited overlapping spatial patterns with task-induced activations for sensory-motor, visual, auditory, attention and other cognitive processing in corresponding brain systems (Table S1 in supporting information). According to previous investigations, these components were the prominent sensory and cognitive networks in resting state [17]–[19]. The sensory networks (the involved brain regions) were SMN (bilateral sensory-motor cortex (SMC) and posterior parietal lobule/precuneus (PPL/PCu)), LVN (bilateral lateral visual cortex (LVC) such as fusiform gyrus and inferior occipital gyrus), MVN (medial visual cortex (MVC) such as primary visual cortex), AN (bilateral temporal lobe (TEM)), and the cognitive networks (the involved brain regions) were the DAN (left superior frontal gyrus (sFG) and left temporoparietal junction (TPJ)), VAN (right medial frontal gyrus/superior frontal gyrus (mFG/sFG) and the right TPJ), DMN (bilateral ventral medial prefrontal cortex (vmPFC), bilateral TPJ and bilateral posterior cingulated cortex (PCC) and SRN (bilateral vmPFC) (see details in the spatial maps and patterns in Figure 1). According to the assessments of connectivity and network strengths, learning induced alterations were proved in the networks shown with red color scale in Figure 1. The DMN of cognitive networks and the LVN and SMN of sensory networks exhibited significant alterations for the experimental group after learning.

**Figure 2:**
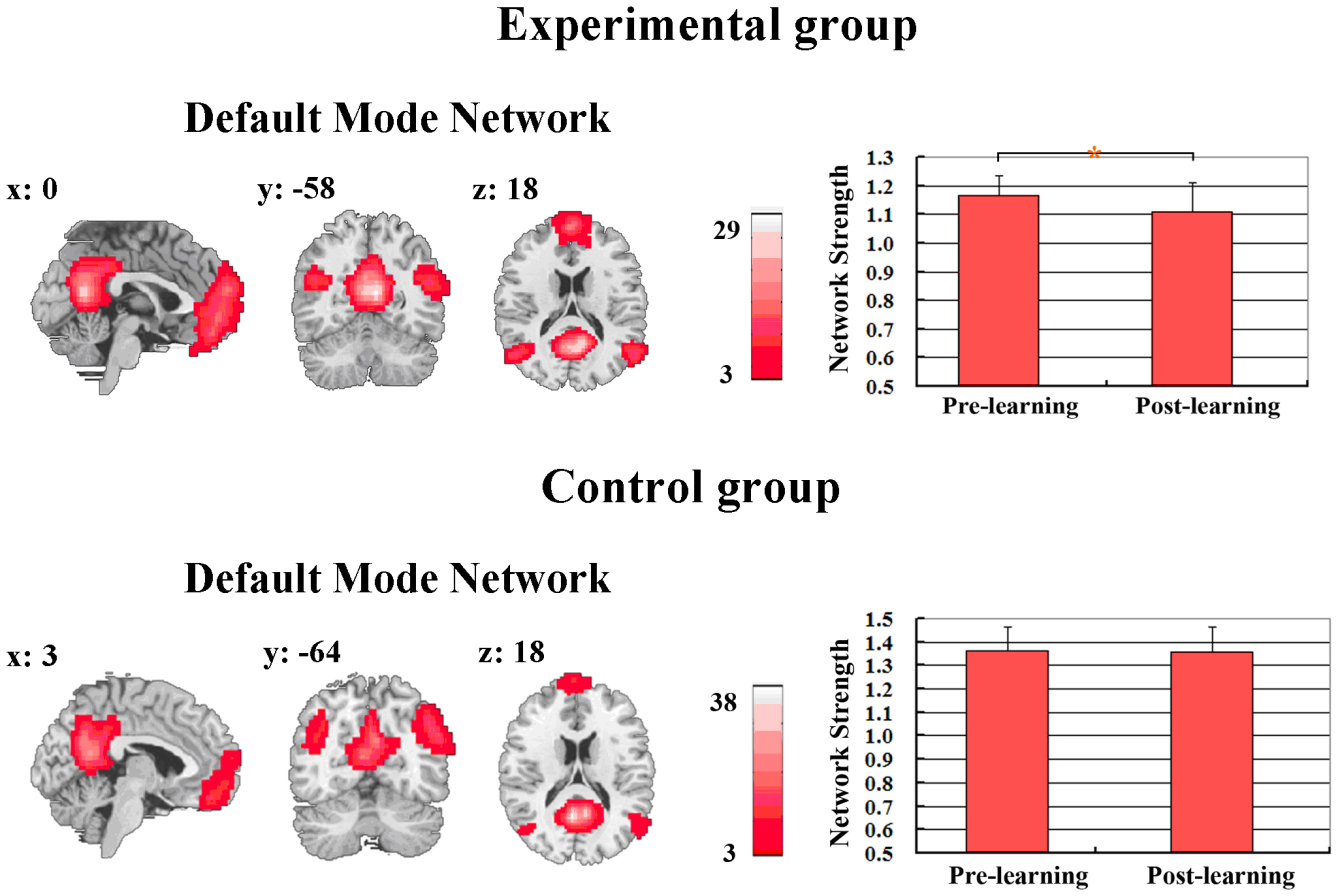
Network strength in the default mode network decreased in the experimental group but not in the control group. The RSNs were identified across all participants in the experimental and control groups respectively. Network strength was assessed based on the integrative spatial map of each RSN and further compared between the rest scans before and after learning for each group. * *p*<0.05.

Depending on the assessment of network strength, learning-induced alterations during resting state were manifested in the cognitive networks but not in the sensory networks. The DMN, as one of the cognitive RSNs, showed a significant decrease in network strength after learning (Figure 2). Such a decrease was only detected in the experimental group but not in the control group (Figures 2a and 2b, *F*(1, 24) = 6.17, *p*<0.05, corrected).

**Figure 3:**
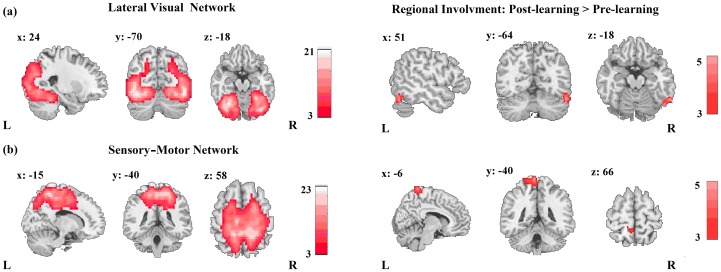
Alterations of connectivity strength only in the experimental group after learning. (a) Alteration of connectivity strength in the lateral visual network. (b) Alterations of connectivity strength in the sensory-motor network. The RSNs were identified across all participants in the experimental and control groups respectively. The connectivity strength was measured statistically based on each voxel in the spatial map of each RSN and further compared between the rest scans before and after learning for each group. The statistical threshold was set at *p*<0.05, corrected for multiple comparisons at the cluster level.

Interestingly, according to the assessment of connectivity strength, only sensory RSNs, specifically the LVN and SMN exhibited significant alterations of connectivity strength during resting state after learning (Figure 3). In the LVN, increased connectivity strength mainly focused on right fusiform gyrus (Figure 3a, Table 1). As to the SMN, the increase of connectivity strength was identified in left precuneus (Figure 3b, Table 1). No significant variations in connectivity strength were detected in the control group when comparing the pre- and post-rest scans (Table 1).

**Table 1:** Regions in RSNs exhibited alterations of the connectivity strength after learning for both experimental and control groups.

RSNs	Regions	L/R	BA	Peak MNI Coordinates			
				x	y	z	t_max_
**Experimental group**							
LVN	Fusiform Gyrus	R	19/37	48	−64	−18	3.90
SMN	Precuneus	L	7	−6	−43	74	4.32
**Control group**							
None							

The statistical threshold was set at *p*<0.05, corrected for multiple comparisons at the cluster level.

## Discussion

The present study stressed resting-state functional connectivity before and after a two-week period of motor imagery learning. Based on previous explorations, we first identified eight sensory and cognitive RSNs corresponding to the brain systems and further explored the functional connectivity of these RSNs through the assessments of connectivity and network strengths before and after the learning. Our results revealed greater connectivity strengths in fusiform gyrus and precuneus of the sensory RSNs, LVN and SMN after learning, which may be associated with the sensory processing of learning in the resting state [7], [31]. Additionally, decreased network strength induced by learning was proved in the cognitive RSN specifically DMN, suggesting the attenuated cognitive processing of learning [32], [33]. These alterations in resting-state functional connectivity after learning potentially subserved the establishment of motor schema and the regulation of introspective thought.

The sensory RSNs, LVN and SMN of the visual and sensory-motor brain systems exhibited significant variances in connectivity strength after learning. Increased connectivity strength was identified in right fusiform of the LVN and left precuneus of the SMN. The LVN was suggested to be involved in visual processing during resting state, and one potential role of LVN may be associated with memory consolidation [34]. Fusiform gyrus of the LVN is a critical region in motor learning, especially in motor imagery learning [3]. In the present study, a prominent role for this area was also discovered during resting state after learning. These findings suggested that the neural plasticity associated with motor imagery learning may be formed in this area, and a role related to visual memory was one of the potential explanations for fusiform gyrus in the LVN during learning [3], [35].

The SMN recruited cortical areas which exhibited significant activations in most sensory-motor tasks, and the spontaneous activities observed in the SMN during resting state have been suggested to subserve coordinated processing in motor tasks [31]. Previous investigations focused on executive learning, and primarily revealed the learning effects in the sensory-motor system during resting state [7], [8], [13], [21]. As to motor imagery learning, our results showed that the SMN exhibited increased connectivity strength in precuneus after learning, and such an increase might be associated with the prominent role of precuneus, illustrated in previous studies, in visuospatial and memory processing [36], [37]. Precuneus potentially coordinated with sensory-motor cortex to establish motor schema through motor imagery learning [36]. During motor imagery learning, visuospatial information could be integrated and internally represented as visual images [12]. After learning, the established motor schema may require further processing such as memory retrieval through precuneus. However, further exploration is still required to fully elucidate this issue.

In the present study, cognitive RSNs specifically the DMN showed significant alterations of network strength after learning. Although the function of the DMN is currently unclear, it still has been considered as one of the critical RSNs [38], [39]. Recent computational evidences showed that the DMN could integrate information from other RSNs [19], [43]. Therefore, a decrease of network strength of the DMN after motor imagery learning may reflect the attenuated integration of information from other RSNs. Furthermore, studies have indicated that the DMN as the default brain system was involved in many cognitive processing, such as remembering the past, planning the future, and mind wandering [40], [41]. These processing mainly focused on the introspective thought during resting state [39], [42]. Then, the attenuated integration of information for the DMN was probably associated with the regulation of introspective thought after learning [40], [44]. Notably, varied functional connectivity underlying resting state was mostly detected in the DMN for mental disorders [39], [45]. Thus, our results further implicated the potential value of motor imagery learning in mental disorders rehabilitation.

Previous investigations implied that neural plasticity underlying motor skill learning mostly focused on a single brain system (the sensory-motor system for execution learning and the visual system for motor imagery learning) [2]–[5]. Intriguingly, our results further revealed that neural plasticity underlying motor skill learning, especially motor imagery learning could be formed in multiple brain systems. In the visual and the sensory-motor systems, the increased connectivity strengths of fusiform gyrus and precuneus indicated that the sensory processing for motor imagery learning mainly relied on specific brain regions during resting state; Furthermore, the decreased network strength was proved in the default system after motor imagery learning, suggesting that the cognitive processing for the learning may be conducted by the integrative default system. Moreover, investigations have suggested that neural plasticity underlying motor skill learning was related to the functional reorganizations and functional connectivity underlying resting state may play critical roles in the sensory and cognitive processing of previous experiences and introspective thoughts [5], [46]–[48]. Then, the alterations of resting-state functional connectivity after motor imagery learning further implicated that these processing for resting state were potentially reorganized in multiple brain systems. Therefore, therapeutic benefits of motor imagery learning may not be limited to the sensory-motor system.

### Summary

In summary, we assessed resting-state functional connectivity before and after two weeks of motor imagery learning. Cognitive and sensory RSNs in multiple brain systems exhibited alterations at functional connectivity level after motor imagery learning. These alterations, manifested as decreased network strength in the DMN and the increased connectivity strength in fusiform gyrus and precuneus of the LVN and SMN, suggested that resting-state functional connectivity could be modulated by motor imagery learning in multiple brain systems. Such modulation may be associated with the functional reorganizations in resting state, further providing new insights into the therapeutic benefits of motor imagery learning. Nevertheless, as an exploratory investigation, the current study has some limitations. For example, the number of subjects participated in this study is low, thus these findings of this study should be further validated with a relatively large number of subjects. Moreover, the functional interactions between regions in each RSN were not fully examined owing to the methodological restriction in our analyses. Future studies stressing these issues will be helpful in understanding the neural plasticity underlying motor imagery learning.

## Supporting Information

Table S1 **Regions significantly recruited in each RSN for experimental group.** The RSNs identified through a single independent component analyses on the combined data of pre- and post-rest scans, were first converted to z-maps for each participant and were further tested to determine group t-maps for the experimental group, *p*<0.005, FDR corrected.
(PDF)

Figure S1 **Baseline tests on the network strength of DMN between the experimental and control groups.** (a) The group mean value of experiment/control group; (b) The individual value of experiment/control group.
(PDF)
