# Supplementary material for: Motor Imagery Learning Modulates Functional Connectivity of Multiple Brain Systems in Resting State
Source: PLoS One. 2014 Jan 17;9(1):e85489. doi: 10.1371/journal.pone.0085489 (PMC3894973; doi:10.1371/journal.pone.0085489)
Supplement: Table S1 — Regions significantly recruited in each RSN for experimental group. The RSNs identified through a single independent component analyses on the combined data of pre- and post-rest scans, were first converted to z-maps for each participant and were further tested to determine group t-maps for the experimental group, p<0.005, FDR corrected. (PDF) [file pone.0085489.s001.pdf]

Table S1. Regions significantly recruited in each RSN for experimental group. The RSNs identified through a single independent component analyses on the combined data of pre- and post-rest scans, were first converted to z-maps for each participant and were further tested to determine group t-maps for the experimental group, $p < 0.005$, FDR corrected.

RSNs	Involved brain regions	L/R	BA	Peak MNI Coordinates			
				x	y	z	T_{max}
LVN	Inferior occipital gyrus/ fusiform gyrus	L	19/20/37	-36	-79	-10	20.91
	Inferior occipital gyrus/ fusiform gyrus	R	19/20/37	33	-76	-14	20.37
MVN	Visual cortex	L/R	17/18	6	-85	-10	20.45
AN	Superior temporal gyrus	L	22/42	-45	-19	6	28.50
	Superior temporal gyrus	R	22/42	57	-4	2	28.71
SMN	Postcentral gyrus,	L	3/4	-30	-34	58	11.52
	Postcentral gyrus	R	3/4	33	-31	54	12.23
	Precentral gyrus	L	6	-30	-10	54	12.40
	Precentral gyrus	R	6	33	-10	54	9.25
	Posterior parietal lobe/ Precuneus	L	7	-12	-55	66	12.69
	Posterior parietal lobe/ Precuneus	R	7	9	-52	62	13.50
SRN	Anterior cingulate	L/R	24/32	-3	38	-6	30.84
	Medial frontal gyrus	L/R	32	6	44	-10	16.38
	Medial frontal gyrus	L	9/10	-6	44	-10	16.11
DAN	Inferior parietal lobule	L	7/39/40	-39	-58	46	24.97
	Inferior parietal lobule	R	39/40	48	-70	38	13.12
	Middle frontal gyrus	L	8	-36	20	42	19.45
	Middle/Superior frontal gyrus	R	8/9	36	20	50	9.20
VAN	Inferior/Middle temporal gyrus	R	20/21	60	-19	-18	14.46
	Superior parietal lobule	R	7/19	33	-73	50	19.29
	Inferior parietal lobule	R	40	51	-55	38	30.62
	Middle frontal gyrus	R	6/8	48	32	34	23.75
DMN	Posterior cingulate cortex	L/R	23/31	9	-55	14	28.28
	Medial prefrontal cortex	L/R	10	-6	56	-2	16.88

	Inferior parietal cortex	L	39	-48	-70	34	11.65
	Inferior parietal cortex	R	39	45	-67	34	12.71
	Temporal parietal junction	L	20/21	-54	-4	-22	8.09
	Temporal parietal junction	R	20/21	60	-1	-26	10.38
	(Para)Hippocampus	L	28/35	-27	-25	-22	5.30
	(Para)Hippocampus	R	28/35	30	-25	-22	5.80
