# Supplementary material for: Motor Imagery Learning Modulates Functional Connectivity of Multiple Brain Systems in Resting State
Source: PLoS One. 2014 Jan 17;9(1):e85489. doi: 10.1371/journal.pone.0085489 (PMC3894973; doi:10.1371/journal.pone.0085489)
Supplement: Figure S1 — Baseline tests on the network strength of DMN between the experimental and control groups. (a) The group mean value of experiment/control group; (b) The individual value of experiment/control group. (PDF) [file pone.0085489.s002.pdf]

(a)

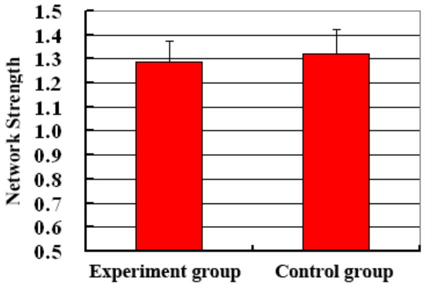

(b)

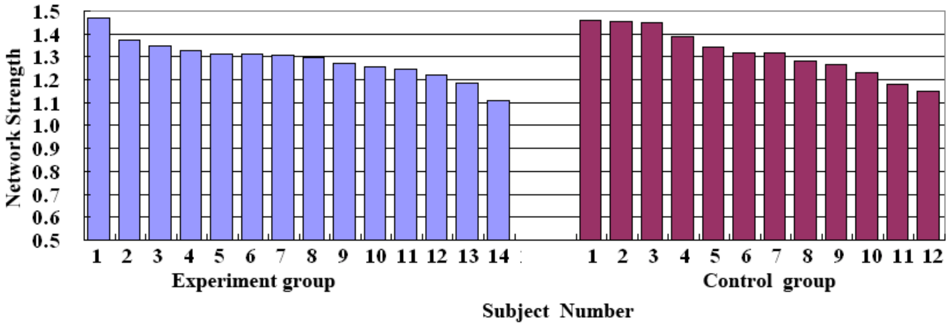

Figure S1. Baseline tests on the network strength of DMN between the experimental and control groups. (a) The group mean value of experiment/control group; (b) The individual value of experimental/ control group.
